# A clinical tool to identify older women with back pain at high risk of osteoporotic vertebral fractures (Vfrac): a population-based cohort study with exploratory economic evaluation

**DOI:** 10.1093/ageing/afac031

**Published:** 2022-03-14

**Authors:** Tarnjit K Khera, Linda P Hunt, Sarah Davis, Rachael Gooberman-Hill, Howard Thom, Yixin Xu, Zoe Paskins, Tim J Peters, Jon H Tobias, Emma M Clark

**Affiliations:** Translational Health Sciences, Bristol Medical School, University of Bristol, Bristol, UK; Translational Health Sciences, Bristol Medical School, University of Bristol, Bristol, UK; School of Health & Related Research, University of Sheffield, Sheffield, UK; NIHR Bristol Biomedical Research Centre, Bristol, UK; Population Health Sciences, Bristol Medical School, University of Bristol, Bristol, UK; Population Health Sciences, Bristol Medical School, University of Bristol, Bristol, UK; Population Health Sciences, Bristol Medical School, University of Bristol, Bristol, UK; School of Medicine, Keele University, Staffordshire, UK; Haywood Academic Rheumatology Centre, Midland Partnership NHS Foundation Trust, Stoke-on-Trent, UK; Translational Health Sciences, Bristol Medical School, University of Bristol, Bristol, UK; Translational Health Sciences, Bristol Medical School, University of Bristol, Bristol, UK; MRC Integrative Epidemiology Unit, University of Bristol, UK; Translational Health Sciences, Bristol Medical School, University of Bristol, Bristol, UK; North Bristol NHS Trust, Bristol, UK

**Keywords:** vertebral fractures, back pain, Osteoporosis, Vfrac, cohort study, older people

## Abstract

**Background:**

osteoporotic vertebral fractures (OVFs) identify people at high risk of future fractures, but despite this, less than a third come to clinical attention. The objective of this study was to develop a clinical tool to aid health care professionals decide which older women with back pain should have a spinal radiograph.

**Methods:**

a population-based cohort of 1,635 women aged 65+ years with self-reported back pain in the previous 4 months were recruited from primary care. Exposure data were collected through self-completion questionnaires and physical examination, including descriptions of back pain and traditional risk factors for osteoporosis. Outcome was the presence/absence of OVFs on spinal radiographs. Logistic regression models identified independent predictors of OVFs, with the area under the (receiver operating) curve calculated for the final model, and a cut-point was identified.

**Results:**

mean age was 73.9 years and 209 (12.8%) had OVFs. The final Vfrac model comprised 15 predictors of OVF, with an AUC of 0.802 (95% CI: 0.764–0.840). Sensitivity was 72.4% and specificity was 72.9%. Vfrac identified 93% of those with more than one OVF and two-thirds of those with one OVF. Performance was enhanced by inclusion of self-reported back pain descriptors, removal of which reduced AUC to 0.742 (95% CI: 0.696–0.788) and sensitivity to 66.5%. Health economic modelling to support a future trial was favourable.

**Conclusions:**

the Vfrac clinical tool appears to be valid and is improved by the addition of self-reported back pain symptoms. The tool now requires testing to establish real-world clinical and cost-effectiveness.

## Key Points

- Vfrac is a clinical tool consisting of 15 questions which can be performed by a practice nurse. The output is a recommendation, or not, for spinal radiographs.
- Vfrac has good sensitivity and specificity for identification of older women with back pain who have OVFs.
- Identification of those with OVFs is improved through the addition of self-reported back pain descriptors.
- Health economic modelling indicates there is potential value in a future randomised controlled trial to evaluate the Vfrac tool.

Osteoporosis and associated fragility fractures are one of the most common musculoskeletal conditions in older people, and approximately three million people in the UK have osteoporosis (www.ons.gov.uk). Osteoporotic vertebral fractures (OVFs) are of particular importance, as they identify people at a high risk of future fracture: within 5 years of the occurrence, 1 in 4 people will have a further vertebral fracture [1], and 1 in 10 will have a limb fracture, including hip fracture [2]. In addition, osteoporotic fractures lead to morbidity and disability: more than a third of patients who experience OVFs have difficulties with activities of daily living for the rest of their lives [3]. However, medications are available to reduce the risk of further vertebral fracture by between 31–65% for bisphosphonates [4], and even greater reductions are possible with anabolic treatments [5]. Despite this, less than a third of people with OVFs come to clinical attention [6]. There are many possible explanations for this diagnostic failure. However, a major reason is a need for knowledge and understanding about which clinical features should trigger referral for diagnostic spinal radiographs in people with possible OVFs [7].

To address this, using the MRC framework for the development and evaluation of complex interventions [8], we developed a clinical decision tool called ‘Vfrac’. The tool helps primary care practitioners decide if an older woman with back pain is at high risk of an OVF and requires a spinal radiograph to confirm the diagnosis.

We carried out four preliminary studies. First, we performed a cross-sectional study of 509 women from primary care [9] and identified four independent predictors of OVF which could be combined into a tool to determine who should have spinal radiographs. A predetermined cut-point gave a sensitivity of 62% and a specificity of 71%. We investigated this prototype tool to identify if it could have utility for clinical decisions in a randomised controlled trial of 3,200 unselected older women from the community [10]. Results showed that allocation to screening approximately doubled the odds of a new prescription for osteoporosis medications (OR: 2.24, 95% CI: 1.16–4.33, *P* = 0.016). However, cost-effectiveness modelling suggested that it was unlikely to be cost-effective from the NHS perspective, mainly because of a low prevalence of OVF in this unselected population. We then focussed on the population of older women with back pain [11] and identified six independent predictors of OVF, four of which were newly identified pain descriptors. Finally, to identify if other symptoms, sensations and pain experiences had been missed, we carried out a qualitative focus group study [12] of 19 older adults with OVFs. Results showed that womens’ experiences of vertebral fractures related to seven sensations, with pain being the dominant one.

The aims of this current study were to: (i) enhance the original prototype tool [10] with the newly identified pain descriptors [10] and other sensations [12] to develop an improved clinical decision tool (Vfrac) for use when older women present to primary care with back pain; (ii) identify the changes in prediction accuracy when including self-reported back pain descriptors over and above traditional risk factors for OVF and (iii) estimate the tool’s potential cost-effectiveness to identify if it is reasonable to conduct further evaluation.

## Methods

The Vfrac cohort study recruited participants from multiple general practices within two areas of the UK: Bristol and Stoke-on-Trent. Research ethics approval was obtained from the National Research Ethics Service (West of Scotland REC 18/WS/0061). All participants provided written consent. The protocol was published before data collection [13]; one substantial amendment was made to allow recruitment of those who had already had a spinal radiograph within the previous 4 months. The study was registered with the ISRCTN Registry (https://doi.org/10.1186/ISRCTN16550671).

### Study design and participants

Thirteen general practices from a range of deprivation scores as assessed by the Index of Multiple Deprivation were recruited from Stoke-on-Trent and nine from Bristol. Women aged ≥65 with a self-reported episode of back pain in the previous 4 months were recruited. For more information, see Supplementary Data, Section 1.

### Exposure data

#### Back pain data

A wide range of questions were included in a self-completion questionnaire (see protocol paper for full description [13]) based on previous studies on women with and without OVFs [11, 14] plus other back pain questionnaires [15, 16]. Findings from the qualitative study [12] were also used to develop questions for quantitative data collection. The Margolis pain diagram [17] was included for participants to mark the anatomical site of their back pain.

#### Other self-reported data

Data were collected on frailty, traditional risk factors for osteoporosis, concomitant illnesses, health-related quality of life, health care usage at baseline and 3 months later and use of pain relieving medication at baseline and 3 months later using the same question structure as previous studies [11, 18]. Fragility fracture was defined as fracture after age 50 excluding hands, feet, head and excluding high trauma.

#### Physical examination

Data collected by a trained research nurse were: height, weight, chest expansion, waist circumference, rib-to-pelvis distance and wall-tragus distance. Reported height loss was calculated by subtracting the height measured in the research clinic from self-reported height at 25 years of age. For more information, see Supplementary Data, Section 1.

### Outcome data: OVFs

All participants had lateral thoracic and lumbar radiographs. Radiographs were assessed for the presence or absence of OVFs by EC using the Algorithm-based qualitative method [19]. Radiographs were categorised into those with no fracture or with fracture. Those with OVFs were further categorised into mild, moderate or severe fractures based on their ‘worst’ fracture using the Genant semi-quantitative method [20]. Repeatability of the primary outcome was assessed by a random sample of anonymised images reviewed by E.C. and an independent experienced radiologist (S.G.) 4 months after the completion of initial data collection. Results showed complete agreement for intra-rater reliability by E.C. The kappa for agreement between E.C. and S.G. was 0.689, indicating substantial agreement. There was 100% agreement between E.C. and S.G. for moderate and severe OVFs.

### Statistical analysis

Preliminary univariable analysis explored relationships between each predictor variable and OVF using logistic regression. Variables found related to OVF with *P* < 0.1 were taken forward to the next stage of the analysis. For this, a series of logistic regression models were carried out using subsets of the predictor variables; this pragmatic approach was adopted as many predictors had missing values. Groups of predictor variables were considered together using backwards stepwise logistic regression analyses to remove those with *P* > 0.1. Age was constrained to stay in the model irrespective of its *P* value. The reduced subsets of predictor variables were then combined and analysed with a similar backwards stepwise approach. Having determined a ‘final’ model, the discarded predictors were added back individually to check that none would further improve the model. Regression coefficients needed to calculate the linear predictor, the maximum likelihood *R*-squared and AUC calculated are reported for the final model obtained. Model validation included calibration-in-the-large, calibration slope and heuristic shrinkage [21]. Five hundred bootstrapped samples were created and were used to estimate shrinkage and adjust the calibration slope and AUC optimism. As the final model was calculated from complete cases, 10 multiply imputed data sets were combined to re-estimate the regression coefficients on the full set. A cut-point of the final linear predictor was identified based on a maximised sum of sensitivity and specificity chosen because this method weighs false negatives and positives equally and is equivalent to minimising Youden’s Index. The added benefit of the use of self-reported symptoms was assessed by looking at the proportions of those identified with OVFs using the cut-point before and after removal of these symptoms.

### Sample size

Full details are available in the protocol paper [13]. The sample size was calculated as 1,633 based on the following assumptions: a prevalence of OVFs between 12 and 20% based on data from the European Vertebral Osteoporosis Study [22], a margin of error of 5% and sensitivity and specificity of the Vfrac tool between 80 and 95%.

### Health economic analyses

Full explanation of the health economic analysis is available in the Supplementary Data, Section 1, Methods. In addition, Supplementary Data, Section 2 describes a within-study analysis, the results of which drove the requirement to move to a modelling-based approach for the economic analysis. The decision tree structure used for modelling is illustrated in Supplementary Data, Section 3. Current standard of care was defined from stakeholder work as consultation with GP for back pain which was followed by potential referral for radiograph. To compare the cost-effectiveness of the Vfrac tool to this standard of care, the proportions of people diagnosed with OVF by the Vfrac tool and by current standard of care were modelled, as were the life-time costs and quality-adjusted-life-years (QALYs). Simulations were used to estimate the expected lifetime costs and QALYs according to whether the individual received treatment with the bisphosphonate alendronic acid, or no treatment, using a previously published bisphosphonate cost-effectiveness model [23]. An NHS and personal social services perspective were adopted for the analysis. For both Vfrac and standard of care groups, life-time net benefits were calculated at a willingness to pay threshold of £20,000/QALY. These were used to calculate the probability that Vfrac or standard of care was most cost-effective (i.e. intervention with greatest net benefit at £20,000/QALY). Expected Value of Perfect Information (EVPI) per person and population EVPI were estimated [24] to measure the value of removing all uncertainty in all parameters.

## Results

A total of 1,635 participants were recruited (see Supplementary Data, Section 4 for STROBE diagram), with a mean age of 73.9 years (range: 65.4–96.8 years). Of these, 209 (12.8%) had VFs: 134 (8.2%) with one and 75 (4.6%) with more than one (range: 2–9). Thirty-four participants were excluded from further analysis (33 due to spinal malignancy/metalwork, 1 due to missing baseline questionnaire), leaving 1,601 (202, 12.6%, with OVF) for the main analysis.

Full data of all univariable analyses are available in the Supplementary Data, Section 5. Initially, univariable analyses were undertaken to look at associations between the individual descriptive words for back pain and the presence or absence of OVF. Backwards stepwise logistic regression analysis identified the strongest determinants of OVF (Supplementary Tables 3 and 4). Similarly, univariable analyses were undertaken to look at the associations between change in back pain with specific activities (Supplementary Table 5), anatomical site of pain (Supplementary Table 6), change in pain over time (Supplementary Table 7) or posture-related pain (Supplementary Table 8) and the presence or absence of OVF. At this stage, the 12 putative pain predictors were combined together, with backwards stepwise analysis used to identify which were the strongest determinants of OVF (Supplementary Table 9). Only back pain described as stinging, described as sharp, described as like toothache, agreement with ‘If I’m working in the kitchen, like chopping vegetables or washing, my back pain gets worse and worse to reach a peak—then I have to sit down immediately’ and pain marked on the Margolis diagram in the thoracic or low back/buttock area were associated with OVF.

Data were collected on whether specific situations increased back pain, decreased back pain or had no effect (Supplementary Table 10). Eight putative predictors were identified, but a multivariable backwards stepwise analysis removed three, leaving bending, sitting on straight backed chairs, sitting on soft chairs, sleeping and changes in the weather.

Subsequent univariable analyses identified frailty variables, walking distance and use of walking aids were associated with the presence or absence of OVF (Supplementary Table 11). Next, univariable associations between the traditional risk factor for osteoporosis and the presence of absence of OVF were assessed, and use of oral steroids for >3 months was identified along with previous fracture (Supplementary Tables 12 and 13). No association was seen between concomitant illnesses and the presence or absence of OVF (Supplementary Table 14).

Finally, data collected during the physical examination were analysed for associations with OVF (Supplementary Table 15). Backwards stepwise analysis removed four variables, leaving weight, wall-to-tragus and height loss as independent predictors of OVF, together with age (Supplementary Table 16).

Backwards logistic regression further reduced the variables from Supplementary Tables 9, 10 and 16 (*n* = 1,490). All variables that had been excluded, either in previous steps or preliminary univariable analyses were then re-assessed. Only steroid use for >3 months approached significance (*P* = 0.052) and, given the well-recognised clinical association between glucocorticoids and OVF, was added back in to produce the final model. Further backwards stepwise removal and some close scrutiny of the resulting models finally led to the inclusion of two extra variables: pain affected by walking and reclining.

**Figure 1:**
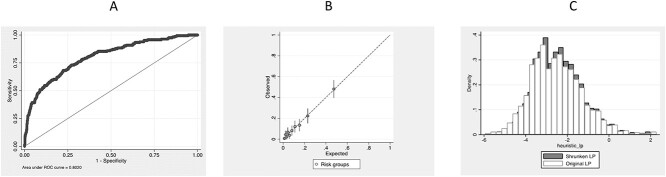
Statistical validation of Vfrac showing: (A) receiver operating characteristic curve illustrating the diagnostic ability of Vfrac to identify those with OVF. The area under the curve is 0.802, 95% CI: 0.764–0.840; (B) a calibration plot over 10 risk groups defined by deciles of the linear predictor and (C) the original linear predictor and the shrunken linear predictor.

**Figure 2:**
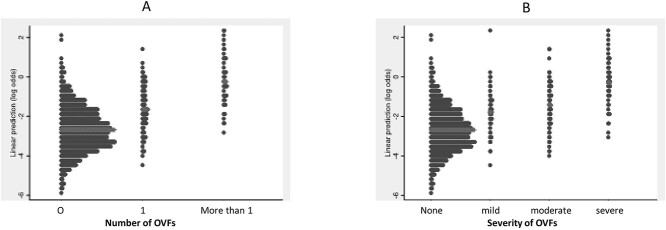
Graphs showing the mean (heavy lighter grey line) and spread of the linear predictor for (A) those with no, one or more than one OVF and (B) for those with no, mild, moderate or severe OVFs.

The final Vfrac model is shown in Table 1. The prevalence of OVF in this final data set was 163/1,337 (12.2%). The mean value of the linear predictor was −2.50 (SD: 1.25), with the mean ± SD linear predictor for those without OVFs being −2.68 ± 1.12 and for those with OVFs being −1.18 ± 1.37. This yielded an AUC of 0.802 (0.764–0.840) (Figure 1A). The calibration slope was 1.0, showing no evidence of overfitting or underfitting (Figure 1B). A heuristic estimate of shrinkage was calculated to be 0.925. This was used to estimate a ‘shrunken’ linear predictor to assess the impact of regression towards the mean in any future real-world use. The mean (SD) of the shrunken linear predictor was −2.43 (SD: 1.16). Figure 1C compares the distributions of the linear predictor and with its the shrunken values. Figure 2 illustrates the separations accorded by the linear predictor between participants with none, one or more than one OVFs (Figure 2A) and none, mild, moderate and severe OVFs (Figure 2B). From 500 bootstrapped samples, optimism in the estimate of the AUC was estimated to be 0.019, therefore the optimism-adjusted AUC was 0.783. Finally, as a secondary analysis to check our results, multiple imputation was used to account for the missing data, with results for the imputed model being similar to those seen in Table 1.

**Table 1:** Final Vfrac model—multivariable independent associations between variables and the presence or absence of vertebral fractures (*n* = 1,337; 163 with OVFs)

	Multivariable odds ratio per unit change in predictor (95% CI), *n* = 1,337	Coefficient (SE), *P*
Age (years)	0.98 (0.94–1.01)	−0.0239 (0.018), *P* = 0.190
Weight (kg)	0.98 (0.96–0.99)	−0.0251 (0.007), *P* = 0.001
Wall to tragus (cm)	1.07 (1.01–1.13)	0.0673 (0.029), *P* = 0.021
Reported height loss (cm)	1.17 (1.10–1.25)	0.1568 (0.032), *P* < 0.001
Pain described as sharp	0.63 (0.40–0.99)	−0.4615 (0.231), *P* = 0.046
Pain described as like toothache	0.49 (0.27–0.91)	−0.7050 (0.311), *P* = 0.024
Agreement with ‘If I’m working in the kitchen like chopping vegetables or washing my back pain gets worse and worse to reach a peak—then I have to sit down immediately’	1.97 (1.30–3.00)	0.6799 (0.213), *P* = 0.001
Pain in thoracic area of Margolis diagram	1.66 (1.11–2.49)	0.5073 (0.206), *P* = 0.014
Pain in low back/buttock area of Margolis diagram	0.64 (0.44–0.94)	−0.4433 (0.196), *P* = 0.024
Pain increased by walking	0.55 (0.37–0.84)	−0.5918 (0.210), *P* = 0.005
Pain affected by sitting on straight-backed chairs	1.78 (1.16–2.74)	0.5779 (0.220), *P* = 0.009
Pain affected by sitting on soft chairs	0.48 (0.32–0.71)	−0.7431 (0.201), *P* < 0.001
Pain increased by reclining	1.93 (1.24–3.02)	0.6588 (0.228), *P* = 0.004
Fracture after age 50, excluding hands, feet, head and excluding high trauma	3.33 (2.30–4.82)	1.2021 (0.189), *P* < 0.001
Steroids for >3 months	1.37 (0.81–2.32)	0.3124 (0.270), *P* = 0.247
Constant		−1.9355 (1.456)

Predictor variables are put into the regression equation by stating with the coefficient, then the first four variables are entered in the units shown multiplied by their specific coefficient. The remainder variables are all coded zero or one, so the regression coefficients for these reflect the amount added or subtracted if the item is reported.

The final cut-point of the linear predictor for identification of which older women with back pain should have a spinal radiograph because of a high risk of fracture was −2.00, chosen as this gave a sensitivity of 72.4% and a specificity of 72.9%. Assuming the same prevalence identified in this study cohort, the final model has a positive predictive value of 27.1% and a negative predictive value of 95.0% (Table 2). Without inclusion of back pain symptoms, the Vfrac tool identifies 66.5% of those with OVFs (53.7% with one; 92.5% with more than one), and sensitivity is reduced to 66.5%. Adding back pain symptoms identifies 72.4% of those with OVFs (62.0% with one OVF; 92.7% with more than one), as shown in Table 2. Removing self-reported symptoms reduces the AUC from 0.802 to 0.742 (95% CI: 0.696–0.788).

**Table 2:** Table illustrating the effect of using a cut-point of −2.00 for the linear predictor

	Linear predictor		
Binary outcome	<−2.00, *N* (%)	≥−2.00, *N* (%)	Total
No VF	856 (72.9%)	318 (27.1%)	1,174 (100%)
VF	45 (27.6%)	118 (72.4%)	163 (100%)
Total	901 (67.4%)	436 (32.6%)	1,337 (100%)
Number of OVFs	<−2.00, *N* (%)	≥−2.00, *N* (%)	Total
No VF	856 (72.9%)	318 (27.1%)	1,174 (100%)
One VF	41 (38.0%)	67 (62.0%)	108 (100%)
More than one VF	4 (7.3%)	51 (92.7%)	55 (100%)
Severity of OVFs	<−2.00, *N* (%)	≥−2.00, *N* (%)	Total
No VF	856 (72.9%)	318 (27.1%)	1,174 (100%)
Mild VFs	20 (41.7%)	28 (58.3%)	48 (100%)
Moderate VFs	21 (33.3%)	42 (66.7%)	63 (100%)
Severe VFs	4 (7.7%)	48 (92.3%)	52 (100%)

Cost-effectiveness results are presented in Table 3. The lifetime incremental net benefit for Vfrac tool compared to standard of care is £1.47 (95% credible interval: −£2,587, £2,456), with 49.4% probability of being cost-effective. The uncertainty translates into a high value in future research with the estimated EVPI being £526 per patient and EVPI per population being £229–458 million, comfortably outweighing the cost of any large-scale randomised controlled trial.

**Table 3:** Cost-effectiveness analyses (mean and 95% credible intervals)

	Standard of care	Vfrac	Incremental Vfrac—standard of care
Proportion cohort with OVF and referred for radiograph	0.025 (0.012, 0.037)	0.091 (0.075, 0.11)	0.066 (0.046, 0.088)
Proportion cohort with OVF and not referred for radiograph	0.10 (0.081, 0.12)	0.034 (0.024, 0.044)	−0.066 (−0.089, −0.046)
Proportion cohort with no OVF but referred for radiograph	0.17 (0.087, 0.25)	0.25 (0.22, 0.27)	0.072 (−0.012, 0.16)
Total costs (£)	315.67 (267.65, 370.99)	322.95 (274.12, 375.08)	7.28 (−58.59, 73.04)
Total QALYs	0.63 (0.53, 0.73)	0.63 (0.53, 0.73)	0.00044 (−0.13, 0.13)
Net benefit (£, at £20,000/QALY)	12,192 (10,227, 14,208)	12,193 (10,344, 14,209)	1.47 (−2,587, 2,456)
Probability of Cost Effectiveness	0.506	0.494	NA
EVPI (£)	526		
Population EVPI (£)	229–458 million		

## Discussion

We now have a clinical tool, Vfrac, to help health care practitioners decide which older women presenting to primary care with back pain are at high risk of currently having one or more OVFs and therefore require a diagnostic spinal radiograph. Of these recommended to have radiographs by Vfrac, approximately one third will have an OVF. Furthermore, Vfrac will identify >90% of those with severe OVFs and approximately two-thirds of those with mild or moderate fractures. The output looks robust and valid. Preliminary modelling suggests there is great uncertainty about the cost-effectiveness of implementing Vfrac, and these findings strongly support a new trial of Vfrac to establish its real-world clinical and cost-effectiveness.

There are simple clinical tools to guide osteoporosis management decisions currently in use within primary care, such as FRAX, which estimate future probability of major osteoporotic and hip fracture (https://www.shef.ac.uk/FRAX/). However, FRAX does not give any information on the risk of existing (prevalent) OVFs. The Vfrac tool is unique, as the only evidence-based decision tool able to highlight an individual who should have a spinal radiograph because of their risk of an existing OVF.

Compared to the original cross-sectional study [9], sensitivity and specificity for identification of people with OVFs are improved with the Vfrac tool, presumably because more detailed information about back pain has been included. In addition, compared to the previous randomised controlled trial in unselected older women, the Vfrac tool identifies a higher proportion of those with moderate and severe OVFs. Finally, patients’ accounts of back pain are necessarily subjective in nature, but our modelling suggests that the use of self-reported back pain descriptors in addition to more traditional risk factors for osteoporosis improves the AUC of Vfrac, particularly for those with one OVF.

There are limitations to this study. The recruited study population is unlikely to be fully representative of the background population. Our study has a shortfall in Asian, Black, Mixed and other ethnicities and, *a priori*, no men were included to restrict the development work to those with a high background prevalence of osteoporosis (women). Vfrac was also designed before the coronavirus pandemic, and much more health care is now delivered virtually by telephone or video, pointing to the need to develop Vfrac as a remote self-completion tool, which is now being tested. As the Vfrac tool is targeted at those presenting with back pain, it cannot identify those with asymptomatic OVF, and real-world testing is required to identify if limiting Vfrac to those with back pain impacts on the clinical- or cost-effectiveness. Finally, to evaluate cost-effectiveness definitively, there needs to be a control comparator. The study was not designed to provide this comparison but to assess whether there were grounds to conduct further evaluation of the tool’s clinical and cost effectiveness.

The Vfrac tool has been designed as a web-based online tool, future-proofed, so it will be supportable through NHS IT systems. The source code can be resurrected into any appropriate format, such as a mobile website or an app, depending on future IT infrastructure. We are now ready to assess real-world clinical and cost-effectiveness of Vfrac to improve the detection of older adults with OVFs and to improve bone health.

## Supplementary Material

aa-21-1454-File002_afac031
