# Supplementary material for: A clinical tool to identify older women with back pain at high risk of osteoporotic vertebral fractures (Vfrac): a population-based cohort study with exploratory economic evaluation
Source: Age Ageing. 2022 Mar 14;51(3):afac031. doi: 10.1093/ageing/afac031 (PMC8918203; doi:10.1093/ageing/afac031)
Supplement: aa-21-1454-File002_afac031 [file aa-21-1454-file002_afac031.docx]

A clinical tool to identify older women with back pain at high risk of osteoporotic vertebral fractures (Vfrac): a population-based cohort study with exploratory economic evaluation

**SUPPLEMENTARY DATA**

**Contents**

Section 1 Pg 2 Supplementary methods

Section 2 Pg 8 Supplementary results: A within-study health economic analysis

Section 3 Pg 12 Supplementary figure: Health economic decision tree

Section 4 Pg 13 Supplementary figure: Vfrac STROBE diagram

Section 5 Pg 14 Supplementary tables 1-16

**SECTION 1: SUPPLEMENTARY METHODS**

Study design and participants

Thirteen general practices from a range of neighbourhoods and deprivation scores as assessed by the Index of Multiple Deprivation (IMD) in 2015, were recruited from Stoke-on-Trent and 9 from Bristol. Practices that took part in the original preparatory^12^ and pilot studies^13,15^ were not recruited. Practices invited all eligible women registered on their system to take part by post between July 2018 and January 2020. Eligibility was solely age 65 or more. Those who reported an episode of back pain in the previous four months completed further data collection through physical examination and spinal radiograph – see Supplementary Section 4, Vfrac STROBE diagram on pg 14. Participant ethnicity was compared with the UK census from 2011, where 86.0% were white and 14.0% a mix of Asian, Black, Mixed and other. In the Vfrac study, 78.5% self-reported as white, 20.7% declined to answer, and 0.9% self-reported as non-white, indicating that the cohort has a shortfall in non-white participants.

Exposure data

The majority of exposure data was self-reported; similar studies using identical data collection methods have generally good agreement with electronic GP records^13^. Specifically for this study, the self-reported co-morbidity data were compared against electronic GP records and results showed good agreement for some measures (e.g. self-reported diabetes 95.8% confirmed), but less for others (e.g. 66.0% for chronic lung disease).

*Back pain data*: A wide range of questions were included in the self-completion questionnaire (see protocol paper for full description^17^) based on previous studies on women with and without OVFs^15,18^ plus other back pain questionnaires^19,20^. Findings from the qualitative study^16^ were also used to develop questions for quantitative data collection that asked whether participants agreed or disagreed with various statements about how back pain changed with activity and other descriptive statements. The Margolis pain diagram^21^ was included to allow study participants to mark the anatomical site of their back pain, and as used in previous studies investigating OVFs^13^, marks in thoracic area, waist area and low back/buttock area were used in this analysis. Where participants were asked to indicate whether they agreed or disagreed whether an activity or posture made their back pain worse but neither had been ticked, these types of missing data were recoded as ‘disagree’. The implications of this were to reduce the strength of any association seen for univariable associations but increase power overall by reducing missing data.

*Other self-reported data*: Data were collected on frailty (walking aid use, walking distance, falls, concomitant illnesses), traditional risk factors for osteoporosis (previous fractures, use of steroid tablets, family history of hip fracture, smoking, and alcohol intake), concomitant illnesses (including diabetes, inflammatory arthritis, anxiety and heart disease), health related quality of life (EQ5D-5L^22^), healthcare usage at baseline and three months later (interactions with healthcare professionals in primary and secondary care) and use of pain killing medication at baseline and three months later using the same question structure as previous research studies^15,23^. Previous fractures were categorised according to age at fracture, anatomical site of fracture and level of trauma of the precipitating injury^24^ if known. A pragmatic decision was taken, based on ease of data collection/recollection whilst attempting to capture previous fragility fractures, to use the following definition: fracture after aged 50 or over excluding hands, feet, head and excluding high trauma (falls from more than 3 metres, car accidents, being hit by a heavy moving object or crushed in a machine).

*Physical examination*: Data were collected during the research clinic on all participants, by trained research nurses, chosen from literature review and our previous research^12,13^ and were: (1) height without shoes and without headgear measured with a free-standing stadiometer; (2) weight without shoes, hats, coats and cardigans/jumpers on a calibrated weighing machine; (3) chest expansion using a validated method^25^; (4) waist circumference using the WHO method^26^; (5) rib-to-pelvis distance using a validated method^27^; and (6) wall-tragus distance using a validated method^28^. Reported height loss was calculated by subtracting the height measured in the research clinic from that recorded on the baseline questionnaire (self-reported height at 25 years of age). Those whose measured height was taller than self-reported height at age 25 years were censored so reported height loss was recorded as zero.

Outcome data: Osteoporotic vertebral fractures (OVFs)

All participants had lateral thoracic and lateral lumbar radiographs. Antero-posterior (AP) views were not performed for pragmatic reasons to reduce radiation exposure and financial cost. Radiographs were assessed for the presence or absence of OVFs by an experienced clinical researcher (EC) using the Algorithm-Based Qualitative (ABQ) method^29^. Radiographs were categorised into those with no fracture and those with fracture. Site and number of fractures were also noted. As a secondary outcome, those with OVFs were further categorised into mild, moderate or severe fractures based on their ‘worst’ fracture using the Genant semi-quantitative (SQ) method^30^. Repeatability of the primary outcome was assessed by a random sample of anonymised images reviewed by EC and an independent experienced radiologist (SG) 4 months after completion of initial data collection. Neither EC nor SG knew the previously assigned categorisation. Results showed complete agreement for intra-rater reliability by EC. The kappa for agreement between EC and SG (inter-rater reliability) was 0.689 indicating substantial agreement. There was 100% agreement between EC and SG for moderate and severe OVFs. EC under-diagnosed mild OVFs compared to SG.

Statistical analysis

Preliminary univariable analysis explored the relationships between each (categorical or continuous) predictor variable in turn using logistic regression. Variables found related to OVF at this stage with P<0.1 were taken forward to the next stage of the analysis. For this, a series of logistic regression models were carried out using subsets of the predictor variables in turn; this pragmatic approach was adopted in part as there was a large number of predictors of which many had missing values and complete case analysis at this stage would have led to a much reduced data set. Moreover, this approach allows greater interpretive control over variable selection, including reducing the risks of multi-collinearity going unnoticed, as well as generally increased epidemiological insights to potentially be gleaned. Groups of predictor variables were considered together using backwards stepwise logistic regression analyses to remove those with P>0.1. Continuous predictor variables were further investigated as to whether the model could be improved by their prior transformation using fractional polynomials up to the second degree. Age was constrained to stay in the model, irrespective of its P value. The reduced subsets of predictor variables were then combined and analysed with a similar backwards stepwise approach. Having determined a ‘final’ model, the discarded predictors were added back individually to check that none would further improve the model, and those that did were added back using likelihood ratio tests to assess improvement. Regression coefficients needed to calculate the linear predictor, the maximum likelihood R-squared, Brier’s score and AUC calculated are reported for the final model obtained. Model validation included calibration-in-the-large (CITL), calibration slope and heuristic shrinkage^31^. Five hundred bootstrapped samples were created and used to estimate shrinkage and adjust the calibration slope and AUC optimism. As the final model was calculated from complete cases, 10 multiply imputed data sets were combined to re-estimate the regression coefficients on the full set of 1,601. Multiple imputation assumed missing at random (MAR). Subgroup analyses were performed to look at results from the final model separately for the two participating centres. A cut-point of the final linear predictor was identified based on a maximised sum of sensitivity and specificity. The added benefit of the use of self-reported symptoms was assessed by looking at the proportions of those identified with OVFs using the cut-point before and after removal of these symptoms. All analyses were carried out using Stata 16.0.

Sample size

Full details are available in the protocol paper^17^. The sample size was calculated as 1633, based on the following assumptions: a prevalence of OVFs between 12-20% based on data from the European Vertebral Osteoporosis Study^32^; a margin of error of 5%, and sensitivity and specificity of the Vfrac tool between 80 and 95%. 1633 will be large enough to encompass and specificity of Vfrac as sample sizes required for specificity are much lower.

Health economic analyses

*Within study analysis:* In Supplementary Section 2, pg 8 of this document, it is shown that more severe patients are referred or consult with their GP and patients always have higher costs and worse EQ-5D profiles at follow-up, regardless of referral or GP consultation. This finding made it difficult to use the Vfrac study data to construct a counterfactual analysis to show any benefit of Vfrac referral for OVF or GP consultation for back pain. Modelling was therefore instead used for the health economic analysis.

*Stakeholder work*: This was undertaken to provide a description of current standard of care and to sense check the baseline data. An online survey was sent to clinicians in the field of osteoporosis and primary care, and to patients written in plain language. Results were anonymous, and questions covered estimations of healthcare usage including consultation rates for back pain and getting a spinal radiograph in the presence of back pain, as well as sense-checking the baseline data collected in the study for healthcare use. Seven clinicians and 12 patients took part.

*Health economic modelling:* The decision tree structure used for modelling is illustrated in Supplementary Section 3 on pg 12. This decision tree was designed in discussion with our clinical team and reflecting on findings of the stakeholder work. The model reflects the consensus that patients with suspected OVF are either referred or not referred for radiograph and that patients diagnosed by radiograph as having OVF are always assigned to treatment. Current standard of care was defined from stakeholder work as consultation with GP for back pain followed by potential referral for radiograph. To compare the cost-effectiveness of the Vfrac tool to this standard of care, the proportions of people diagnosed with OVF by the Vfrac tool and by current standard of care were modelled, as were the life-time costs and quality-adjusted-life-years (QALYs). Model parameters are described here briefly but full details are provided in Supplementary Section 5, Supplementary Tables 1 and 2 on pg 15 and 16. For patients receiving standard-of-care, we assumed all patients to have a GP consultation with the proportion referred being 20% with a 95% credible range of 10-30%, modelled by a Normal distribution, following the stakeholder work. For patients receiving Vfrac, it was important to distinguish not only whether the patients had an OVF, but also whether patients would be recommended for radiograph by the Vfrac tool. Proportions with OVF referred for radiograph, with OVF but not referred for radiograph and without OVF but referred for radiograph were modelled with a Dirichlet distribution with parameters equal to the numbers of patients in these categories in the Vfrac study. Uncertainty was modelled using 1000 samples from these distributions. The cost of radiograph (£72) was taken from standard NHS costs and no radiograph disutility was applied.

Life-time costs and QALYs for OVF diagnosed (i.e. treated with standard bisphosphonate anti-fracture medication) and OVF undiagnosed (i.e. untreated) were obtained using a previously published bisphosphonate cost-effectiveness model^33^. This long-term model was developed to inform appraisals of osteoporosis treatments by the UK’s National Institute for Health and Care Excellence and therefore the methods used comply with the reference case for cost-effectiveness analysis in the UK. It uses a discrete event simulation (DES) framework. The key clinical events modelled are fractures at the hip, vertebrae, wrist or proximal humerus, all-cause mortality, fracture-related mortality, and new admission to long-term residential care following hip fracture. Costs are estimated from an NHS and Personal Social Services perspective and QALYs are estimated using utility values derived from the UK valuation of the EQ-5D. Patient characteristics for simulated patients were set to match the distribution in the 118 patients in the study with a positive Vfrac score and an OVF. These had an average age of 76 years (SD 6.3). Repeated resampling from this cohort of 118 yielded a cohort of 50,000 patients. The model was run 1000 times for this cohort of 50,000 using different parameter samples each time, averaging the heterogeneity. Simulations were used to estimate expected lifetime costs and QALYs according to whether the individual received treatment with the bisphosphonate alendronic acid, or no treatment. Lifetime costs and QALYs from this model are summarised in Supplementary Table 2 on pg 16.

Total costs and QALYs for Vfrac and standard of care were calculated and summarized with mean and Bayesian 95% credible intervals (CrI); these are the Bayesian equivalent of frequentist confidence intervals. For both life-term Vfrac and standard of care groups, net benefits were calculated at a willingness to pay threshold of £20,000/QALY, the current standard for UK healthcare decision making. These were used to calculate the probability that Vfrac or standard of care was most cost-effective (i.e. intervention with greatest net benefit at £20,000/QALY). Expected Value of Perfect Information (EVPI) per person and population EVPI were estimated^34^, to measure the value of removing all uncertainty in all parameters (i.e. those listed in Supplementary Table 1 on pg 15and the life-term costs and QALYs from the bisphosphonate model in Supplementary Table 2 on pg 16). Population EVPI is an upper bound on the value of a randomized controlled trial on the efficacy of the Vfrac diagnostic tool. A time-horizon for the population EVPI of 10 years was applied with discounting at 3.5% per year. The population was that of women aged ≥65 years in the UK consulting with their GP for back pain (further details in Supplementary Table 1 on pg 15).

The key assumptions of the health economic analysis are:

1. The consultation time for nurses is an average of 9.72 minutes.
2. For costing painkillers, if the patient takes painkillers on a few days/week we assume their medication cost is the weighted average daily drug cost multiplied by a random number drawn from a normal distribution ranging from 1 to 3.5 (i.e. incurs day of drug costs 1-3.5 days a week); if the patient takes painkillers at least once/twice per day, we assume their medication cost is the weighted average daily drug cost multiplied by a random number drawn from a normal distribution ranging from 3.5 to 7 (i.e. incurs daily drug cost 3.5 to 7 days per week). These weekly frequencies were multiplied by average daily cost to get 7-day costs and then, assuming 30-day months. (Shown in Supplementary HE Table 3 on pg 10)
3. For patients receiving standard of care, we assumed all patients to have a GP consultation with the proportion referred being 20% with a 95% credible range of 10-30%, modelled by a Normal distribution, following the stakeholder work.
4. Distribution assumption in the proportion of different patient types for the Vfrac health economic modelling (Shown in Supplementary Table 1 on pg 15)

Patient and Public Involvement

Patients and the public have been involved since 2012 in planning and delivery of the research projects that led up to development of the Vfrac tool. Two people with OVFs provided input into the steering committee of the study. Our award-winning Patient Experience Partnership in Research (PEP-R)^35^ helped to write the patient/public facing paperwork including Patient Information Leaflets for recruitment, an infographic for communication of results, and plain language summaries for the next stages of Vfrac testing.

**SECTION 2: WITHIN-STUDY HEALTH ECONOMIC (HE) ANALYSIS**

In this appendix we explain **why it is not possible to use the Vfrac data to construct a counterfactual analysis to show any benefit of Vfrac diagnosis or GP consultation, as more severe patients are referred or consult with their GP and patients always become worse at follow-up regardless of referral or consultation.**

**METHODS**

**We describe here the methods used to calculate the total costs and quality adjusted life years (QALYs) for patients with and without osteoporotic vertebral fracture (OVF) who would or would not have been referred for radiograph, and therefore diagnosed, by the Vfrac tool. We also describe methods to calculate these costs and QALYS for a hypothetical standard of care consisting only of GP consultations of patients with back-pain for lateral thoracic and lateral lumbar radiographs.**

**Through the Vfrac programme, 1635 older women with back pain in the previous 4 months were recruited from the community and data collection was based on self-reporting and physical examination. The data available for each patient includes clinical characteristics (e.g. whether or not radiograph shows an OVF), whether they would be referred for radiograph by the Vfrac tool, whether they had a GP consultation for back-pain in the past 3 months, resource use and Euroqol 5-dimensions questionnaire 5-level (EQ5D-5L) at baseline, plus 3 month follow up data on resource use and EQ5D-5L. The analysed sample consisted of only 1600 women as 35 patients did not report baseline EQ5D-5L or medication data.**

**Resource use**

**Resource use (i.e. costs) recorded were medical professional consultations (i.e. healthcare usage) and painkiller medication taken.**

**For consultations, we multiplied the unit cost of each consultation by the number of those consultations per patient over a three-month period in the questionnaire, with the sum over all types giving the cost of consultations. Unit costs were obtained from the Personal Social Services Research Unit (PSSRU) 2020 and the NHS website, and these are summarised in HE Table 1. For nurse consultations, consultation time is required. ^1, 2^ A recent study reported this as being on average 9.72 minutes. ^3^ We assume that the consultation time for nurse types is an average of 9.72 minutes. In addition, the unit costs of Osteopath and Chiropractor are zero from the NHS perspective as neither service is covered by the NHS.**

HE Table 1 Unit costs for different types of consultation and data sources.

| **Consultation type** | **Unit cost (£)** | **Data sources** |
| --- | --- | --- |
| **GP** | **28** | **Unit costs health and social care 2020** |
| **Nurse** | **6.156** | £38/hour from PSSRU and average 9.72min/consultation from Hobbs, et al. (2016) |
| **Hospital specialist** | **117** | Unit costs health and social care 2020 |
| **Physio** | **55** | Unit costs health and social care 2020 |
| **Osteopath** | **0** | Not reimbursed by the NHS |
| **Chiropractor** | **0** | Not reimbursed by the NHS |
| **OT** | **106** | Unit costs health and social care 2020 |
| **Orthotics** | **6.156** | unit costs health and social care 2020 |
| **Pharmacist** | **0** | Community pharmacists are not paid for by the NHS. |

**Daily medication costs came from the British National Formulary (BNF).^4^ Medication costs were a weighted average of daily drug costs, with weights based on the proportion of patients receiving each type of medication in the Vfrac study shows in HE** HE Table 2**.**

HE Table 2 Daily cost of drugs and weighted average daily drug costs from Vfrac study data.

| Drugs types | Quantity | Quantity Unit | Assumption on daily dose | Daily Costs(£) | Proportion  (n=1634*) |
| --- | --- | --- | --- | --- | --- |
| Paracetamol 500mg tablets | 100 | Tablet | Full standard daily dose | 0.0536 | 36.2% |
| Ibuprofen 400mg tablets | 168 | Tablet | Full standard daily dose | 0.968 | 12.1% |
| Co-codamol 15mg/500mg tablets | 100 | Tablet | Full standard daily dose | 0.074 | 8.7% |
| Codeine 30mg tablets | 100 | Tablet | 50% standard daily dose | 0.3512 | 8.7% |
| Amitriptyline 10mg tablets | 56 | Tablet | 10mg per day | 0.0461 | 7.1% |
| Citalopram 20mg tablets | 28 | Tablet | Full standard daily dose | 0.0257 | 6.1% |
| Aspirin 75mg gastro-resistant tablets | 28 | Tablet | Full standard daily dose | 0.0429 | 5.3% |
| Ibuprofen 10% gel | 100 | Gram | A tube lasts a month /31 days a month | 0.1226 | 5.1% |
| Weighted average daily drug costs | £0.187 | | | | |

***Sample size for prescription costs was 1634 as one patient did not complete baseline questionnaire. The 1600 patients used in health economic analysis reported both baseline resource use and EQ5D-5L.**

**Total cost of medication was calculated using reported frequency of taking painkiller medication. Patients were asked "How often have you taken pain killers for your back pain in the last week?" and given four options: 0=I do not take painkillers, 1= on a few days/week, 2=once/twice per day, 3=3-4 times per day, and 4=4-6 times per day. We categorised these responses into numbers of painkillers taken per week as explained in HE** HE Table 3**. If the patient does** not take painkillers**, then their medication cost is 0; if the patient takes** painkillers on **a few days/week, then their medication cost is the weighted average daily drug cost multiplied by a random number from a normal distribution ranging from 1 to 3.5 (i.e. incurs day of drug costs 1-3.5 days a week); if the patient takes** painkillers at least once/twice **per day, then their medication cost is the weighted average daily drug cost multiplied by a random number from a normal distribution ranging from 3.5 to 7 (i.e. incurs daily drug cost 3.5 to 7 days per week). These weekly frequencies were multiplied by average daily cost (HE** HE Table 2**) to get 7-day costs and then, assuming 30-day months, scaled to 3-month medication costs.**

HE Table 3 Assumption on weekly drug does costs calculation.

| Response category* | Assumed frequency | Distribution |
| --- | --- | --- |
| 0 | 0 | 0 |
| 1 | Incurs daily drug costs 1 and 3.5 days per week. | Normal mean 2.25 and standard deviation 0.638 |
| ≥2 | Incurs daily drug costs 3.5 and 7 days per week. | Normal mean 5.25 and standard deviation 0.638 |

***0=I do not take painkillers, 1= on a few days/week, 2=once/twice per day, 3=3-4 times per day, and 4=4-6 times per day**

**EQ5D-5L score and QALYs**

**Baseline and 3-month follow-up EQ5D-5L questionnaire were completed by patients in the Vfrac study. In this case, these were converted to utility scores using the** EuroQoL value set dated 19^th^ January 2019.^5^ **Baseline** and follow-up quality adjusted life years (QALYs) were calculated by multiplying their EQ5D-5L score by 0.25 (i.e. 3 months).

**Total costs and QALYs on Vfrac and those with GP consultation**

Whether a patient would be referred for lateral thoracic and lateral lumbar radiographs by the Vfrac tool and whether they had an OVF was recorded. We could therefore tabulate total cost and QALYs across these categories of patients.

It was also recorded if a patient had a GP consultation in the past 3 months for back pain. **The study did not record if a patient was referred for radiograph by GP consultation in the past 3 months so this could not be used as a hypothetical “standard of care” comparator for the Vfrac tool. For the long-term analysis (as described in the main text), we used stakeholder work on the proportion of patients consulting with a GP for back pain who would be referred for radiograph. In the tabulation of total cost and QALYs using Vfrac data, we only categorise patients based on having or not having a GP consultation for backpain, and do not model the hypothetical proportion referred for radiograph.**

**RESULTS**

**The estimated costs and QALYs are provided in HE Table 4 and HE Table 5, respectively. The key finding is that costs are always higher at follow-up than at baseline and QALYs are lower at follow-up than at baseline for all categories of patient except those without GP consultation and without OVF. In patients with OVF, those with GP consultation have higher costs and lower QALYs than those without, indicating that more severe patients go to their GPs.**

**Similarly, patients who would have been referred for radiograph by Vfrac have higher costs and lower QALYs than those who would not have been referred, suggesting the Vfrac tool picks up more severe patients. The consequence of these findings is that it is not possible to use the Vfrac data to construct a counterfactual analysis to show any benefit of Vfrac diagnosis or GP consultation, as more severe patients are referred or consult with their GP and patients always become worse at follow-up regardless of referral or consultation.**

HE Table 4 Mean costs (£) (95% reference range) using Vfrac study data in patients with or without GP consultation, hypothetically referred for radiograph by Vfrac or not, and with or without osteoporotic vertebral fracture (OVF)*

|  | **OVF** | | **No OVF** | |
| --- | --- | --- | --- | --- |
|  | **Baseline** | **Follow-up** | **Baseline** | **Follow-up** |
|  | **£ cost (95% ref range)** | **£ cost (95% ref range)** | **£ cost (95% ref range)** | **£ cost (95% ref range)** |
| **All patients** | **90.58 (0.00, 377.85)** | **95.61 (0.00, 383.76)** | **73.85 (0.00, 413.60)** | **79.11 (0.00, 422.35)** |
| **Patients with GP consultation** | **136.53(0.92, 380.11)** | **143.26 (11.89, 384.38)** | **72.85 (14.50, 145.53)** | **72.85 (14.50, 145.53)** |
| **Patients without GP consultation** | **85.96 (0.00, 363.94)** | **90.91 (0.00, 368.10)** | **87.15 (0.00, 366.99)** | **92.13 (0.00, 377.58)** |
| **Patients who would have been referred for radiograph by Vfrac** | **94.01 (0.00, 383.18)** | **98.49 (0.00, 395.71)** | **119.63 (0.00, 775.80)** | **124.88 (0.00, 786.92)** |
| **Patients who would not have been referred radiograph by Vfrac** | **80.56 (0.00, 390.94)** | **84.69 (0.00, 391.28)** | **85.28 (0.00, 365.51)** | **90.29 (0.00, 371.95)** |

*95% reference range is an interval containing 95% of samples.

Table 5 Mean QALYs (95% reference range) using Vfrac study data in patients with or without GP consultation, hypothetically referred for radiograph by Vfrac or not, and with or without osteoporotic vertebral fracture (OVF)*

|  | **OVF** | | **No OVF** | |
| --- | --- | --- | --- | --- |
|  | **Baseline** | **Follow-up** | **Baseline** | **Follow-up** |
|  | **Cost (95% ref range)** | **Cost (95% ref range)** | **Cost (95% ref range)** | **Cost (95% ref range)** |
| **All patients** | **0.15 (0.02, 0.25)** | **0.14 (0.01, 0.24)** | **0.16 (0.03, 0.23)** | **0.16 (0.05, 0.25)** |
| **Patients with GP consultation** | **0.13 (0.02, 0.24)** | **0.12 (0.02, 0.25)** | **0.19 (0.15, 0.21)** | **0.12 (0.01, 0.25)** |
| **Patients without GP consultation** | **0.16 (0.04, 0.25)** | **0.15 (0.02, 0.25)** | **0.15 (0.01, 0.25)** | **0.15 (0.01, 0.25)** |
| **Patients who would have been referred for radiograph by Vfrac** | **0.16 (0.04, 0.25)** | **0.15 (0.04, 0.23)** | **0.15 (0.04, 0.24)** | **0.14 (0.04, 0.20)** |
| **Patients who would not have been referred radiograph by Vfrac** | **0.17 (0.00, 0.22)** | **0.16 (0.00, 0.25)** | **0.15 (0.00, 0.23)** | **0.14 (0.00, 0.23)** |

*95% reference range is an interval containing 95% of samples.

**References**

1. NHS NHS Choices. [www.nhs.uk/conditions/](https://d.docs.live.net/4898e5cc837f6cd5/Documents/Bristol/vfrac%20health%20economics/Publication/www.nhs.uk/conditions/).

2. PSSRU Unit Costs of Health and Social Care 2020. [www.pssru.ac.uk/project-pages/unit-costs/unit-costs-2020/](https://d.docs.live.net/4898e5cc837f6cd5/Documents/Bristol/vfrac%20health%20economics/Publication/www.pssru.ac.uk/project-pages/unit-costs/unit-costs-2020/).

3. Hobbs, F. D. R.; Bankhead, C.; Mukhtar, T.; Stevens, S.; Perera-Salazar, R.; Holt, T.; Salisbury, C., Clinical workload in UK primary care: a retrospective analysis of 100 million consultations in England, 2007-14. *Lancet* **2016,** *387* (10035), 2323-2330.

4. BNF drug. <https://bnf.nice.org.uk/drug/>.

5. EQ-5D EuroQoL Value Set 19Jan2019. euroqol.org/eq-5d-instruments/eq-5d-5l-about/valuation-standard-value-sets/.

**SECTION 3: SUPPLEMENTARY FIGURE**

**Supplementary Figure**: Illustration of decision tree model used for health economic comparison of Vfrac and Standard of Care (SoC)*

* Cohort is assigned to either Vfrac or SoC. They either have underlying osteoporotic vertebral fracture (OVF) or not. Vfrac and SoC have some probability of referring patients for radiograph; for Vfrac these probabilities come from the Vfrac study while for SoC these come from stakeholder work. All referred patients incur a radiograph cost of £72. OVF patients who are referred receive alendronate/bisphosphonate while OVF patients without referral receive no treatment. A lifetime horizon for patients with starting age 76 (chosen to match study data) was adopted. Lifetime QALYs and costs for OVF patients on alendronate or no treatment come from a previously published bisphosphonate model. Patients without OVF do not incur costs or QALYs beyond the radiograph and are assumed zero for calculation (this has no impact on incremental results and thus conclusions). Mean costs and QALYs are presented only for illustration as the model is fully probabilistic.

**SECTION 4: VFRAC STROBE DIAGRAM**


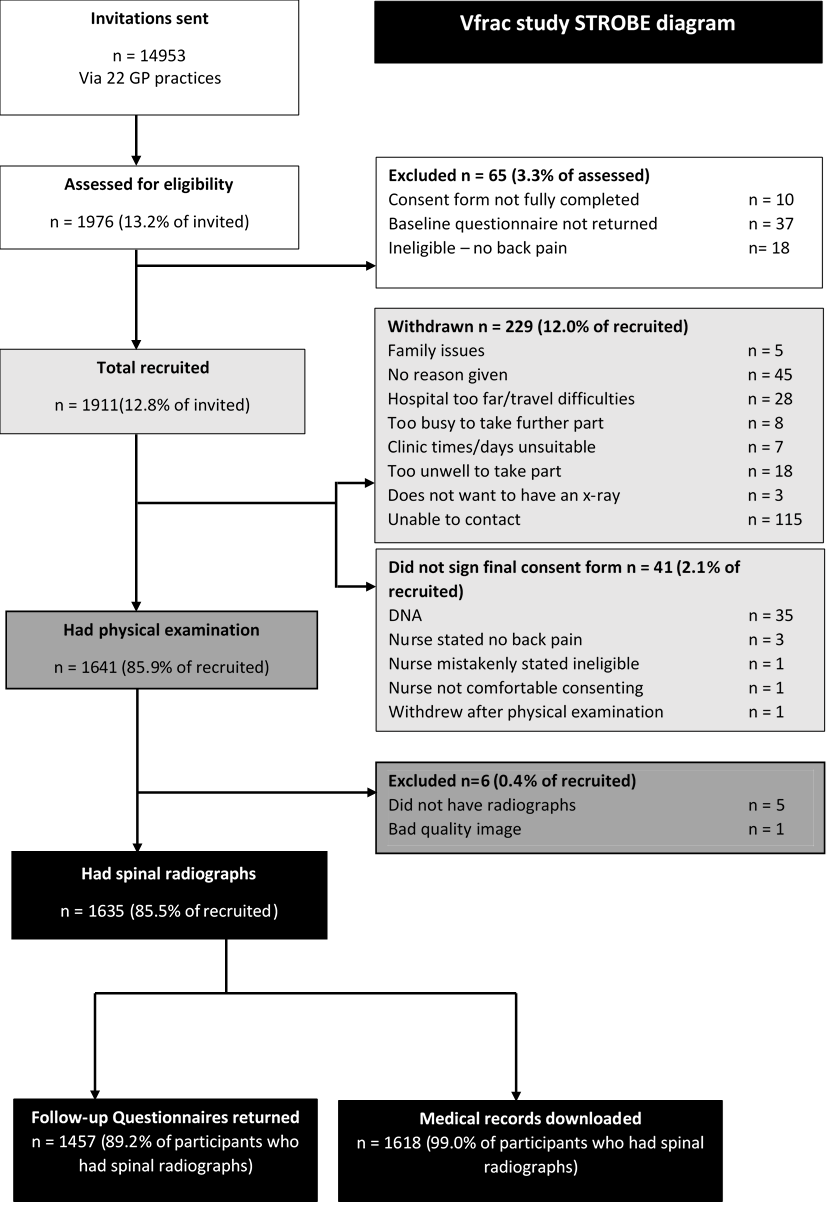


**SECTION 5: SUPPLEMENTARY TABLES**

**Supplementary Table 1:** Distribution assumption in the proportion of different patient types for the Vfrac health economic modelling

| **Parameter** | **Distribution** | **Mean and 95% credible interval** |
| --- | --- | --- |
| Proportion of patients referred for radiograph following GP consultation | Normal (mean=0.2, standard deviation=0.05) | 0.2 (0.11, 0.30) |
| Proportion of patients with OVF | Beta (147, 1183)* | 0.11 (0.09, 0.13) |
| Proportion of patients with OVF referred for radiograph by Vfrac tool | First element of Dirichlet (107, 40, 290, 746)** | 0.091 (0.075 ,0.11) |
| Proportion of patients with OVF not referred for radiograph by Vfrac tool | Second element of Dirichlet (107, 40, 290, 746)** | 0.034 (0.024 ,0.044) |
| The proportion patients without OVF referred for radiograph by Vfrac tool | Third element of Dirichlet (107, 40, 290, 746)** | 0.25 (0.22 ,0.27) |
| **Size of population to benefit for value of information analysis population** | | |
| Number of people aged 65+ in England and Wales^1^ | 12,390,000 | |
| Percentage female of population 65+ in England and Wales^2^ | 51% | |
| Prevalence of back pain consultation among women aged ≥65 years^3^ | 800 to 1600 per 100,000 | |
| Total population over 10 years discounted at 3.5% | 435,129 to 870,258 | |
|  | costs | disutility |
| Radiograph§ | £72 | 0 |

*Alpha and beta of the beta distribution given by numbers of patients in each category in Vfrac study data.

** Parameters given by numbers of patients in each category in Vfrac study data, with common Dirichlet distribution across categories to capture correlation. 1183 patients in total with 746 remaining in category without OVF and without referral by Vfrac.

§ Standard NHS costs

**Supplementary Table 2:** Cost and QALYs estimated by the bisphosphonate model. (Mean with 95% credible interval)

|  | **Costs (£)** | **QALYs** |
| --- | --- | --- |
| **No treatment** | 2436.43 (2277.28, 2601.36) | 5.037 (4.882, 5.186) |
| **Alendronate** | 2394.18 (2225.38, 2597.55) | 5.041 (4.884, 5.189) |
| **Incremental Alendronate - no treatment** | -42.26 (-123.14, 86.23) | 0.00405 (0.000152, 0.00748) |

**Supplementary Table 3**: Back pain descriptives (words) based on McGill pain questionnaire and qualitative focus group work used by participants according to presence of any OVF (n=1596)

| **Word** | **Word not used** | | **Word used** | | **(Univariable)**  **Odds ratio (95%CI)**  **for ‘word used’**  **(vs ‘not used’ as ref.)** |
| --- | --- | --- | --- | --- | --- |
|  | **Number**  **not using** | **Number (%) with OVF** | **Number**  **using** | **Number (%) with OVF** |  |
| **Crushing** | 1,513 | 190 (12.6%) | 83 | 12 (14.5%) | 1.18 (0.63-2.21) P=0.613 |
| **Heavy** | 1,350 | 168 (12.4%) | 246 | 34 (13.8%) | 1.13 (0.76-1.68) P=0.551 |
| **Dull** | 1,016 | 141 (13.9%) | 580 | 61 (10.5%) | 0.73 (0.53-1.00) P=0.053 |
| **Aching** | 289 | 35 (12.1%) | 1,307 | 167 (12.8%) | 1.06 (0.72-1.57) P=0.758 |
| **Sharp** | 1,158 | 168 (14.5%) | 438 | 34 (7.8%) | 0.50 (0.34-0.73) P<0.001 |
| **Agonizing** | 1,354 | 171 (12.6%) | 242 | 31 (12.8%) | 1.02 (0.67-1.53) P=0.938 |
| **Unbearable** | 1,399 | 171 (12.2%) | 197 | 31 (15.7%) | 1.34 (0.89-2.03) P=0.166 |
| **Gnawing** | 1,048 | 135 (12.9%) | 548 | 67 (12.2%) | 0.94 (0.69-1.29) P=0.708 |
| **Excruciating** | 1,444 | 184 (12.7%) | 152 | 18 (11.8%) | 0.92 (0.55-1.54) P=0.751 |
| **Intense** | 1,170 | 151 (12.9%) | 426 | 51 (12.0%) | 0.92 (0.65-1.29) P=0.620 |
| **Stinging** | 1,526 | 189 (12.4%) | 70 | 13 (18.6%) | 1.61 (0.87-3.00) P=0.131 |
| **Cold** | 1,567 | 201 (12.8%) | 29 | 1 (3.5%) | 0.24 (0.03-1.79) P=0.165 |
| **Tingle** | 1,491 | 189 (12.7%) | 105 | 13 (12.4%) | 0.97 (0.53-1.77) P=0.930 |
| **Icy** | 1,588 | 201 (12.7%) | 8 | 1 (12.5%) | 0.99 (0.12-8.05) P=0.989 |
| **Toothache** | 1,328 | 182 (13.7%) | 268 | 20 (7.5%) | 0.51 (0.31-0.82) P=0.006 |
| **Burning** | 1,375 | 174 (12.7%) | 221 | 28 (12.7%) | 1.00 (0.65-1.53) P=0.995 |
| **Brief** | 1,504 | 191 (12.7%) | 92 | 11 (12.0%) | 0.93 (0.49-1.78) P=0.835 |
| **Tiring** | 826 | 98 (11.9%) | 770 | 104 (13.5%) | 1.16 (0.86-1.56) P=0.324 |
| **Niggling** | 1,035 | 133 (12.9%) | 561 | 69 (12.3%) | 0.95 (0.70-1.30) P=0.752 |
| **Continuous** | 1,095 | 142 (13.0%) | 501 | 60 (12.0%) | 0.91 (0.66-1.26) P=0.580 |
| **Annoying** | 922 | 128 (13.9%) | 674 | 74 (11.0%) | 0.77 (0.56-1.04) P=0.085 |
| **Radiating** | 1,274 | 164 (12.9%) | 322 | 38 (11.8%) | 0.91 (0.62-1.32) P=0.605 |
| **Pins&needles** | 1,420 | 184 (13.0%) | 176 | 18 (10.2%) | 0.77 (0.46-1.28) P=0.305 |

**Supplementary Table 4:** Multivariable relationships between back pain descriptives and presence or absence of OVF (n=1596)

| **Word** | **Odds ratio (95%CI)**  **for ‘word used’**  **(vs ‘not used’ as ref.)** |
| --- | --- |
| **Dull** | 0.72 (0.52-0.99) P=0.044 |
| **Sharp** | 0.48 (0.32-0.70) P<0.001 |
| **Stinging** | 1.86 (0.99-3.52) P=0.056 |
| **Toothache** | 0.55 (0.34-0.89) P=0.016 |

**Supplementary Table 5**: Variables describing change in back pain with specific activities and the presence or absence of OVFs (n=1601)

|  | **Disagree/**  **Didn’t indicate** | | **Agree** | | **(Univariable)**  **Odds ratio (95%CI)**  **(vs ‘disagree’ as ref.)** | |
| --- | --- | --- | --- | --- | --- | --- |
|  | **Total n** | **Number (%) with OVF** | **Total n** | **Number (%) with OVF** |  |  |
| When I start doing an activity the pain builds, and builds until it’s agony and I have to stop | 801 | 85 (10.6%) | 800 | 117 (14.6%) | 1.44 (1.07-1.94), P=0.016 |  |
| Walking generally makes my back pain better whilst I’m walking | 950 | 128 (13.5%) | 651 | 74 (11.4%) | 0.82 (0.61-1.12), P=0.213 |  |
| Walking generally makes my back pain better once I’ve finished walking | 1139 | 148 (13.0%) | 462 | 54 (11.7%) | 0.89 (0.64-1.24), P=0.476 |  |
| If I’m working in the kitchen, like chopping vegetables or washing, my back pain gets worse and worse to reach a peak – then I have to sit down immediately | 670 | 54 (8.1%) | 931 | 148 (15.9%) | 2.16 (1.55-3.00), P<0.001 |  |
| If I have to stand for a long time I just know my back pain is going to get worse and worse | 354 | 37 (10.5%) | 1247 | 165 (13.2%) | 1.31 (0.90-1.91), P=0.165 |  |
| Generally my back pain is better with activity | 1009 | 138 (13.7%) | 592 | 64 (10.8%) | 0.77 (0.56-1.05), P=0.096 |  |
| Generally my back pain builds with activity | 743 | 80 (10.8%) | 858 | 122 (14.2%) | 1.37 (1.02-1.86), P=0.039 |  |

**Supplementary Table 6**: Anatomical site of back pain and the presence or absence of OVFs (n=1593)

|  | **Not marked** | | **Yes, marked** | | **(Univariable)**  **Odds ratio (95%CI)**  **(vs ‘not marked’ as ref.)** |
| --- | --- | --- | --- | --- | --- |
|  | **Total n** | **Number (%) with OVF** | **Total n** | **Number (%) with OVF** |  |
| Thoracic area (either left or right or both) | 1211 | 134 (11.1%) | 382 | 68 (17.8%) | 1.74 (1.27-2.39), P=0.001 |
| Waist area (either left or right or both) | 442 | 52 (11.8%) | 1151 | 150 (13.0%) | 1.12 (0.80-1.57), P=0.496 |
| Low back/buttock area (either left or right or both) | 467 | 85 (18.2%) | 1126 | 117 (10.4%) | 0.52 (0.38-0.71), P<0.001 |

**Supplementary Table 7**: Change in pain over time and the presence or absence of OVFs

|  | **Not ticked** | | **Yes, ticked** | | **(Univariable)**  **Odds ratio (95%CI)**  **(vs ‘not ticked’ as ref.)** | |
| --- | --- | --- | --- | --- | --- | --- |
|  | **Total n** | **Number (%) with OVF** | **Total n** | **Number (%) with OVF** |  |  |
| **(A) How pain changes with time (n=1,575)** |  |  |  |  |  |  |
| Continuous, steady, constant | 910 | 112 (12.3%) | 665 | 86 (12.9%) | 1.06 (0.78-1.43), P=0.712 |  |
| Rhythmic, periodic, intermittent | 966 | 122 (12.6%) | 609 | 76 (12.5%) | 0.99 (0.73-1.34), P=0.930 |  |
| Brief, momentary, transient | 1438 | 177 (12.3%) | 137 | 21 (15.3%) | 1.29 (0.79-2.11), P=0.309 |  |
| Other pattern | 1388 | 177 (12.8%) | 187 | 21 (11.2%) | 0.87 (0.54-1.40), P=0.556 |  |
|  |  |  |  |  |  |  |
| **(B) Pictorial description of course of pain (n=1,582)** |  |  |  |  |  |  |
| Persistent pain with slight fluctuations (diagram) | 1130 | 137 (12.1%) | 452 | 62 (13.7%) | 1.15 (0.84-1.59), P=0.388 |  |
| Persistent pain with pain attacks (diagram) | 1315 | 172 (13.1%) | 267 | 27 (10.1%) | 0.75 (0.49-1.15), P=0.184 |  |
| Pain attacks without pain between (diagram) | 1084 | 137 (12.6%) | 498 | 62 (12.5%) | 0.98 (0.71-1.35), P=0.916 |  |
| Pain attacks with pain between (diagram) | 1425 | 185 (13.0%) | 157 | 14 (8.9%) | 0.66 (0.37-1.16), P=0.148 |  |
| Pain increases and increases | 1442 | 176 (12.2%) | 140 | 23 (16.4%) | 1.41 (0.88-2.27), P=0.152 |  |
| Other pattern | 1499 | 188 (12.5%) | 83 | 11 (13.3%) | 1.07 (0.55-2.05), P=0.849 |  |

**Supplementary Table 8**: Posture-related back pain and the presence or absence of OVFs (n=1601)

|  | **I generally disagree**  **/Didn’t indicate** | | **I generally agree** | | **(Univariable)**  **Odds ratio (95%CI)**  **(vs ‘disagree as ref.)** |
| --- | --- | --- | --- | --- | --- |
|  | **Total n** | **Number (%) with OVF** | **Total n** | **Number (%) with OVF** |  |
| It feels as though my upper body is being tugged forward | 1119 | 131 (11.7%) | 482 | 71 (14.7%) | 1.30 (0.95-1.78), P=0.095 |
| It feels as though I am being pulled over all the time | 1324 | 157 (11.9%) | 277 | 45 (16.3%) | 1.44 (1.01-2.07), P=0.046 |
| It feels as though my head is too heavy | 1206 | 156 (12.9%) | 395 | 46 (11.7%) | 0.89 (0.62-1.26), P=0.503 |
| It feels as though there is nothing to hold my head or upper body up | 1307 | 167 (12.8%) | 294 | 35 (11.9%) | 0.92 (0.63-1.36), P=0.684 |

**Supplementary Table 9:** Multivariable relationships between pain variables and the presence or absence of OVFs (n=1588)

|  | **Odds ratio per unit change in predictor, and 95%CI** | **P value** |
| --- | --- | --- |
| Back pain described as stinging | 1.72 (0.90-3.30) | P=0.100 |
| Back pain described as sharp | 0.50 (0.33-0.74) | P=0.001 |
| Back pain described as like toothache | 0.53 (0.33-0.87) | P=0.011 |
| Agreement with ‘If I’m working in the kitchen, like chopping vegetables or washing, my back pain gets worse and worse to reach a peak – then I have to sit down immediately’ | 2.13 (1.53-2.98) | P<0.001 |
| Pain in thoracic area | 1.55 (1.11-2.16) | P=0.010 |
| Pain in low back/buttock area | 0.59 (0.43-0.82) | P=0.001 |

**Supplementary Table 10**: Variables describing situations that affect back pain and the presence or absence of OVFs (n=1601)

|  | **Total n** | **Number (%) with OVF** | **(Univariable)**  **Odds ratio (95%CI)**  **(vs ‘no effect’ as ref.)** |
| --- | --- | --- | --- |
| Walking  No effect  Decrease  Increase | 414  480  707 | 61 (14.7%)  50 (10.4%)  91 (12.9%) | 1  0.67 (0.45-1.00)  0.85 (0.60-1.21)  *Overall P (likelihood ratio) P=0.144* |
| Bending  No effect  Decrease  Increase | 363  82  1156 | 54 (14.9%)  5 (6.1%)  143 (12.4%) | 1  0.37 (0.14-0.96)  0.81 (0.58-1.13)  *Overall P (likelihood ratio) P=0.065* |
| Standing  No effect  Decrease  Increase | 279  63  1259 | 31 (11.1%)  7 (11.1%)  164 (13.0%) | 1  1.00 (0.42-2.39)  1.20 (0.80-1.80)  *Overall P (likelihood ratio) P=0.632* |
| Stretching  No effect  Decrease  Increase | 734  415  452 | 95 (12.9%)  45 (10.8%)  62 (13.7%) | 1  0.82 (0.56-1.19)  1.07 (0.76-1.51)  *Overall P (likelihood ratio) P=0.409* |
| Cold  No effect  Decrease  Increase | 993  30  578 | 136 (13.7%)  1 (3.3%)  65 (11.3%) | 1  0.22 (0.03-1.61)  0.80 (0.58-1.09)  *Overall P (likelihood ratio) P=0.073* |
| Damp  No effect  Decrease  Increase | 998  14  589 | 137 (13.7%)  1 (7.1%)  64 (10.9%) | 1  0.48 (0.06-3.73)  0.77 (0.56-1.05)  *Overall P (likelihood ratio) P=0.198* |
| Lifting  No effect  Decrease  Increase | 309  9  1286 | 45 (14.6%)  0  157 (12.2%) | 1  0.82 (0.57-1.17), P=0.271 |
| Sitting on straight-backed chairs  No effect  Decrease  Increase | 558  524  519 | 55 (9.9%)  69 (13.2%)  78 (15.0%) | 1  1.39 (0.95-2.02)  1.62 (1.12-2.34)  *Overall P (likelihood ratio) P=0.032* |
| Slouching  No effect  Decrease  Increase | 623  104  874 | 88 (14.1%)  13 (12.5%)  101 (11.6%) | 1  0.87 (0.47-1.62)  0.79 (0.58-1.08)  *Overall P (likelihood ratio) P=0.340* |
| Sitting on soft chairs  No effect  Decrease  Increase | 653  239  709 | 101 (15.5%)  27 (11.3%)  74 (10.4%) | 1  0.70 (0.44-1.10)  0.64 (0.46-0.88)  *Overall P (likelihood ratio) P=0.017* |
| Pulling shoulders back  No effect  Decrease  Increase | 937  429  235 | 116 (12.4%)  57 (13.3%)  29 (12.3%) | 1  1.08 (0.77-1.52)  1.00 (0.65-1.54)  *Overall P (likelihood ratio) P=0.889* |
| Sleeping  No effect  Decrease  Increase | 711  467  423 | 99 (13.9%)  64 (13.7%)  39 (9.2%) | 1  0.98 (0.70-1.38)  0.63 (0.42-0.93)  *Overall P (likelihood ratio) P=0.041* |
| Lying down  No effect  Decrease  Increase | 513  652  418 | 74 (13.9%)  87 (13.3%)  41 (9.8%) | 1  0.95 (0.68-1.33)  0.67 (0.45-1.01)  *Overall P (likelihood ratio) P=0.115* |
| Heat  No effect  Decrease  Increase | 776  782  43 | 107 (13.8%)  92 (11.8%)  3 (7.0%) | 1  0.83 (0.62-1.12)  0.47 (0.14-1.54)  *Overall P (likelihood ratio) P=0.233* |
| Using a hot water bottle or electric blanket in bed  No effect  Decrease  Increase | 814  761  26 | 104 (12.8%)  94 (12.4%)  4 (15.4%) | 1  0.96 (0.71-1.30)  1.24 (0.42-3.67)  *Overall P (likelihood ratio) P=0.888* |
| Reclining  No effect  Decrease  Increase | 796  497  308 | 96 (12.1%)  59 (11.9%)  47 (15.3%) | 1  0.98 (0.70-1.39)  1.31 (0.90-1.91)  *Overall P (likelihood ratio) P=0.312* |
| Changes in the weather  No effect  Decrease  Increase | 1049  43  509 | 143 (13.6%)  8 (18.6%)  51 (10.0%) | 1  1.45 (0.66-3.18)  0.71 (0.50-0.99)  *Overall P (likelihood ratio) P=0.063* |
| Twisting  No effect  Decrease  Increase | 539  65  997 | 73 (13.5%)  8 (12.3%)  121 (12.1%) | 1  0.90 (0.41-1.95)  0.88 (0.65-1.20)  *Overall P (likelihood ratio) P=0.731* |

**Supplementary Table 11**: Frailty variables and the presence or absence of OVFs

|  | **Total n** | **Number (%) with OVF** | **(Univariable)**  **Odds ratio (95%CI)** |
| --- | --- | --- | --- |
| Walking distance (n=1,337)  >400 yards  ≤400 yards | 985  352 | 113 (11.5%)  53 (15.1%) | 1  1.37 (0.96-1.94), P=0.081 |
| Use of walking aid (n=1,584)  No  Yes | 1114  470 | 121 (10.9%)  78 (16.6%) | 1  1.63 (1.20-2.22), P=0.002 |
| Falls (n=1,555)  Rarely  Few times per year or more | 1332  223 | 165 (12.4%)  27 (12.1%) | 1  0.97 (0.63-1.50), P=0.906 |

**Supplementary Table 12**: Traditional risk factors for osteoporosis and the presence or absence of OVFs

|  | **Total n** | **Number (%) with OVF** | **(Univariable)**  **Odds ratio (95%CI)** |
| --- | --- | --- | --- |
| Oral steroids for >3 months (n=1,502)  No  Yes | 1344  158 | 154 (11.5%)  32 (20.3%) | 1  1.96 (1.29-3.00), P=0.002 |
| Mother or father had hip fracture (n=1,428)  No  Yes | 1179  249 | 147 (12.5%)  35 (14.1%) | 1  1.15 (0.77-1.71), P=0.495 |
| Smoking (n=1,589)  Never  Gave up  Current | 874  650  65 | 114 (13.0%)  74 (11.4%)  12 (18.5%) | 1  0.86 (0.63-1.17)  1.51 (0.78-2.91)  *Overall P (likelihood ratio) P=0.239* |
| Alcohol intake (n=1,583)  <3 units or less per day  ≥3 units per day | 1436  147 | 177 (12.3%)  23 (15.7%) | 1  1.32 (0.82-2.12), P=0.250 |

**Supplementary Table 13**: Previous fractures and the presence or absence of OVFs

|  | **No** | | **Yes** | | **(Univariable)**  **Odds ratio (95%CI)**  **(vs ‘no’ as ref.)** |
| --- | --- | --- | --- | --- | --- |
|  | **Total n** | **Number (%) with OVF** | **Total n** | **Number (%) with OVF** |  |
| Any fracture after aged 50 | 1007 | 82 (8.1%) | 512 | 103 (20.1%) | 2.84 (2.08-3.88), P<0.001 |
| Fracture after aged 50 excluding hands, feet, head | 1090 | 88 (8.1%) | 437 | 96 (22.0%) | 3.21 (2.34-4.39), P<0.001 |
| Osteoporotic fractures* only after aged 50 | 1436 | 134 (9.3%) | 120 | 53 (44.2%) | 7.69 (5.14-11.49), P<0.001 |
| Any fracture after aged 50 excluding high trauma | 1022 | 85 (8.3%) | 483 | 99 (20.5%) | 2.84 (2.08-3.89), P<0.001 |
| Fracture after aged 50 excluding hands, feet, head excluding high trauma^∞^ | 1106 | 90 (8.1%) | 411 | 93 (22.6%) | 3.30 (2.41-4.53), P<0.001 |
| Osteoporotic fractures* only after aged 50 excluding high trauma^∞^ | 1442 | 137 (9.5%) | 110 | 49 (44.6%) | 7.65 (5.05-11.59), P<0.001 |
| Low trauma^§^ fracture after aged 50 | 1081 | 90 (8.3%) | 424 | 93 (21.9%) | 3.09 (2.26-4.24), P<0.001 |
| Low trauma^§^ fracture after aged 50 excluding hands, feet, head | 1158 | 96 (8.3%) | 357 | 86 (24.1%) | 3.51 (2.55-4.84), P<0.001 |
| Low trauma^§^ osteoporotic fractures only after aged 50 | 1459 | 144 (9.9%) | 92 | 42 (45.7%) | 7.67 (4.92-11.97), P<0.001 |

*Osteoporotic fractures were defined according to anatomical site (hip, vertebral, humeral, forearm)

^∞^High trauma was defined according to the modified Landin description i.e. falls from more than 3 meters, car accidents, being hit by a heavy moving object or crushed in a machine

^§^Low trauma was defined according to the modified Landin description i.e. fall from standing height or less

**Supplementary Table 14**: Concomitant illnesses and the presence or absence of OVFs (n=1594)

|  | **No** | | **Yes** | | **(Univariable)**  **Odds ratio (95%CI)**  **(vs ‘no’ as ref.)** |
| --- | --- | --- | --- | --- | --- |
|  | **Total n** | **Number (%) with OVF** | **Total n** | **Number (%) with OVF** |  |
| Poor vision | 1422 | 179 (12.6%) | 172 | 22 (12.8%) | 1.02 (0.63-1.64), P=0.940 |
| Poor balance | 1034 | 135 (13.1%) | 560 | 66 (11.8%) | 0.89 (0.65-1.22), P=0.466 |
| Menopause before 45 | 1206 | 160 (13.3%) | 388 | 41 (10.6%) | 0.77 (0.54-1.11), P=0.164 |
| Inflammatory arthritis | 1190 | 153 (12.9%) | 404 | 48 (11.9%) | 0.91 (0.65-1.29), P=0.610 |
| Depression | 1181 | 157 (13.3%) | 413 | 44 (10.7%) | 0.78 (0.55-1.11), P=0.165 |
| Memory problems | 1347 | 167 (12.4%) | 247 | 34 (13.8%) | 1.13 (0.76-1.68), P=0.552 |
| Anxiety | 1039 | 139 (13.4%) | 555 | 62 (11.2%) | 0.81 (0.59-1.12), P=0.207 |
| Diabetes (type 1 or 2) | 1429 | 185 (13.0%) | 165 | 16 (9.7%) | 0.72 (0.42-1.24), P=0.236 |
| COPD | 1485 | 187 (12.6%) | 109 | 14 (12.8%) | 1.02 (0.57-1.83), P=0.939 |
| Heart disease | 1424 | 177 (12.4%) | 170 | 24 (14.1%) | 1.16 (0.73-1.83), P=0.531 |

**Supplementary Table 15**: Age and physical examination measurements of participants according to presence of any OVF.

|  | **No OVF** | | | **OVF** | | | **Mann-Whitney U-test to compare the two groups** |
| --- | --- | --- | --- | --- | --- | --- | --- |
|  | **Mean (SD)** | **Median (IQR)** | **n** | **Mean (SD)** | **Median (IQR)** | **n** | **P value** |
| Age (years) | 73.6 (5.6) | 72.5 (69.2-76.8) | 1400 | 76.1 (6.5) | 75.1 (71.2-80.8) | 202 | P<0.001 |
| Height (cm) | 159.0 (6.2) | 158.9 (154.8-163.4) | 1392 | 156.9 (6.6) | 157.5 (152.6-161.8) | 202 | P<0.001 |
| Weight (kg) | 72.7 (15.1) | 70.6 (61.9-80.8) | 1389 | 68.2 (15.4) | 65.3 (57.1-75.4) | 202 | P<0.001 |
| Chest expansion (cm) | 3.4 (1.7) | 3.0 (2.1-4.3) | 1388 | 3.1 (1.6) | 3.0 (2.0-4.0) | 201 | P=0.011 |
| Waist circumference (am) | 93.4 (13.4) | 92.5 (83.5-102.1) | 1391 | 91.4 (13.4) | 89.5 (81.0-101.0) | 202 | P=0.028 |
| Rib to pelvis distance (number of fingers) | 2.4 (0.9) | 2 (2-3) | 1392 | 2.1 (1.0) | 2 (1-3) | 202 | P<0.001 |
| Wall to tragus distance (cm) | 14.8 (3.5) | 14.0 (12.1-16.8) | 1393 | 16.5 (4.1) | 16.0 (13.4-19.0) | 202 | P<0.001 |
| Reported height loss (cm) | 4.0 (3.0) | 3.6 (1.8-5.7) | 1314 | 6.4 (4.1) | 5.4 (3.5-9.3) | 193 | P<0.001 |

**Supplementary Table 16**: Multivariable relationships between physical examination variables and the presence or absence of OVFs (n=1501)

|  | **Odds ratio per unit change in predictor, and 95%CI** | **P value** |
| --- | --- | --- |
| Age (years) | 1.00 (0.97-1.03) | P=0.965 |
| Weight (kg) | 0.98 (0.96-0.99) | P<0.001 |
| Wall to tragus distance (cm) | 1.07 (1.02-1.13) | P=0.003 |
| Reported height loss (cm) | 1.16 (1.10-1.22) | P<0.001 |

1. Clark D. Population of the UK 2019, by age group. https://www.statista.com/statistics/281174/uk-population-by-age/.

2.

[https://www.ethnicity-facts-figures.service.gov.uk/uk-population-by-ethnicity/demographics/male-and-female-populations/latest]( https://www.ethnicity-facts-figures.service.gov.uk/uk-population-by-ethnicity/demographics/male-and-female-populations/latest)

3. Jordan, K.P., et al., International comparisons of the consultation prevalence of musculoskeletal conditions using population-based healthcare data from England and Sweden. Annals of Rheumatic Diseases,2014. 73: p. 212-218.
